# A Treatise on the Diseases of Infants, Founded on Recent Clinical Observations and Investigations in Pathological Anatomy, Made at the Hospice Des Enfans-Trouvés

**Published:** 1839-11-23

**Authors:** 


*A Treatise on the Diseases of Infants, founded on recent Clinical Observations and Investigations in Pathological Anatomy, made at the Hospice des Enfans-Trouvés.* By C. M. Billard, Docteur en Médecine de la Faculté de Paris, etc. etc. With Notes, by Dr. Ollivier, of Angers. Translated from the Third French Edition, with an Appendix, by James Stewart, M. D. New-York: 1839. 8vo., pp. 620.

The two institutions of Paris which are specially devoted to the treatment of the diseases of children, have given rise to many dissertations upon these affections. One of the hospitals for children is appropriated to those above the age of two years. The other is the Foundling Hospital, which is almost exclusively given up to children who have not completed their first year of life,—and by far the largest proportion of these are much younger, and are admitted but a few days after birth. To the Foundling Hospital, Dr. Billard, the author of the present work, was attached, as resident pupil, or interne.

The internes of the French hospitals are selected amongst the most distinguished pupils, and do not generally enter those designed for children until they have already served a year or two in the hospitals for adults; they are, therefore, young physicians who are well instructed, and of considerable experience. Nevertheless, the treatises which they publish often bear internal marks of a partial and immature method of observation. They are generally excellent works, replete with facts, but the conclusions from these facts are often imperfect. Hence the practitioner is sometimes surprised at the disproportionate importance attached to particulars which are, in themselves, of little moment, and he is apt to be dissatisfied with the comparative feebleness of the practical portion of the work.

Billard's work is strangely tinged with the prevailing tendency of the Parisian system of medicine. That is, it is extremely anatomical, perhaps almost ultra in its mode of investigation, and refers a multitude of diseases to lesions of organs, which are sometimes quite unimportant, or, at any rate, are evidently the mere results of general disorders of the economy. Their trace is left, after death, on the organs of the body; but their actual progress was marked only by the functional disorders which cease with life. Is this an evil or vicious mode of study? It is, if the reader, or, still more, the observer, confounds the effects with the cause; if he identifies too closely disease with the consequences of disease; but if he merely assumes the lesion as a fixed point from which to commence the study of diseased action, by proceeding backwards towards the origin of the disorder, and then grouping together the various lesions which are obviously related together as effects of the same cause, the apparent evil becomes a sound and rational method of observation.

Besides, many disorders are obviously anatomical; that is, the anatomical lesion is the direct cause of death, and destroys life, either from the injured part being in itself essential to the due working of the human machine, or from its clogging the wheels, or removing the supply of active power required for a more important organ. This is especially the case with many of the disorders of the air passages, which are either directly fatal, because their seat is in the lungs, or destroy life by attacking the larynx and trachea, and preventing a due supply of air from reaching these organs. Hence the study of these disorders is totally unintelligible without direct reference to a lesion which is the cause of death, although not, properly speaking, the cause of the disease.

Although the scope and bearing of Dr. Billard's work is, perhaps, rather too exclusively anatomical, the symptoms are detailed with as much care as was practicable under the circumstances in which the children are placed at the Foundling Hospital. These are such, that some classes of symptoms are more conveniently studied there than elsewhere, but those which are only to be observed by the watchful eye of a parent, necessarily escape notice at a hospital, especially a foundling hospital. Thus many physiological points connected with the pulse, the cry, the mode of respiration, &c., are readily detected by an observer who sees the child for a short time in each day, but the varying features in the physiognomy of the child, its uneasiness, and its distaste or craving for a food, are almost beyond the reach of observation in a large institution.

There are, however, a large number of symptoms, as well as lesions, which are much more easily studied in groups of children than in single individuals, and the distinguishing excellence of Dr. Billard's work consists in the very elaborate manner in which he has turned the Foundling Hospital to account, and has decided many points which must always have been left in doubt, if the children had not been grouped together.

The peculiar facilities of the hospital, and the able manner in which they have been made the most of by Dr. Billard, will render the book useful to the practitioner; not that it will supply the place of a complete work on the diseases of children, but because it furnishes much information that cannot be obtained elsewhere, and the physician who exercises his own powers of reflection, will know how to combine two different classes of facts in such a way as to render them of practical utility. We have stated that the work is too strictly anatomical, and in that we have pointed out its leading error. The practitioner will not, however, fail to observe another which might seem of cardinal importance—that is, the treatment. The truth is, that the position of the children at all Foundling Hospitals is such as to place them under the worst possible hygienic circumstances. Treatment, therefore, may be judicious, but it will never be sufficient to counteract the influence of injurious circumstances which are constantly acting upon the child, and rendering the efforts of the therapeutist almost nugatory. The work, therefore, should not be regarded as a model of successful treatment; it furnishes a foundation for treatment, and nothing more was probably designed by the author, who was quite conscious of the inherent deficiencies of the Foundling Hospital.

Of the translation itself we may speak in the most favourable terms; it is carefully executed, and as free from gallicisms as the difficulty of the task will allow.

One fault is extremely inconvenient to the reader, and might readily have been avoided: it is the want of distinct separate headings for each page. A work, which will be frequently consulted, like that of Billard, should present every facility for reference.

